# Supplementary material for: Aphid Resistance Segregates Independently of Cardenolide and Glucosinolate Content in an Erysimum cheiranthoides (Wormseed Wallflower) F2 Population
Source: Plants (Basel). 2024 Feb 6;13(4):466. doi: 10.3390/plants13040466 (PMC10893121; doi:10.3390/plants13040466)
Supplement: Supplementary file 1 [file plants-13-00466-s001.zip › Erysimum-aphid Supplemental Figures.pdf]

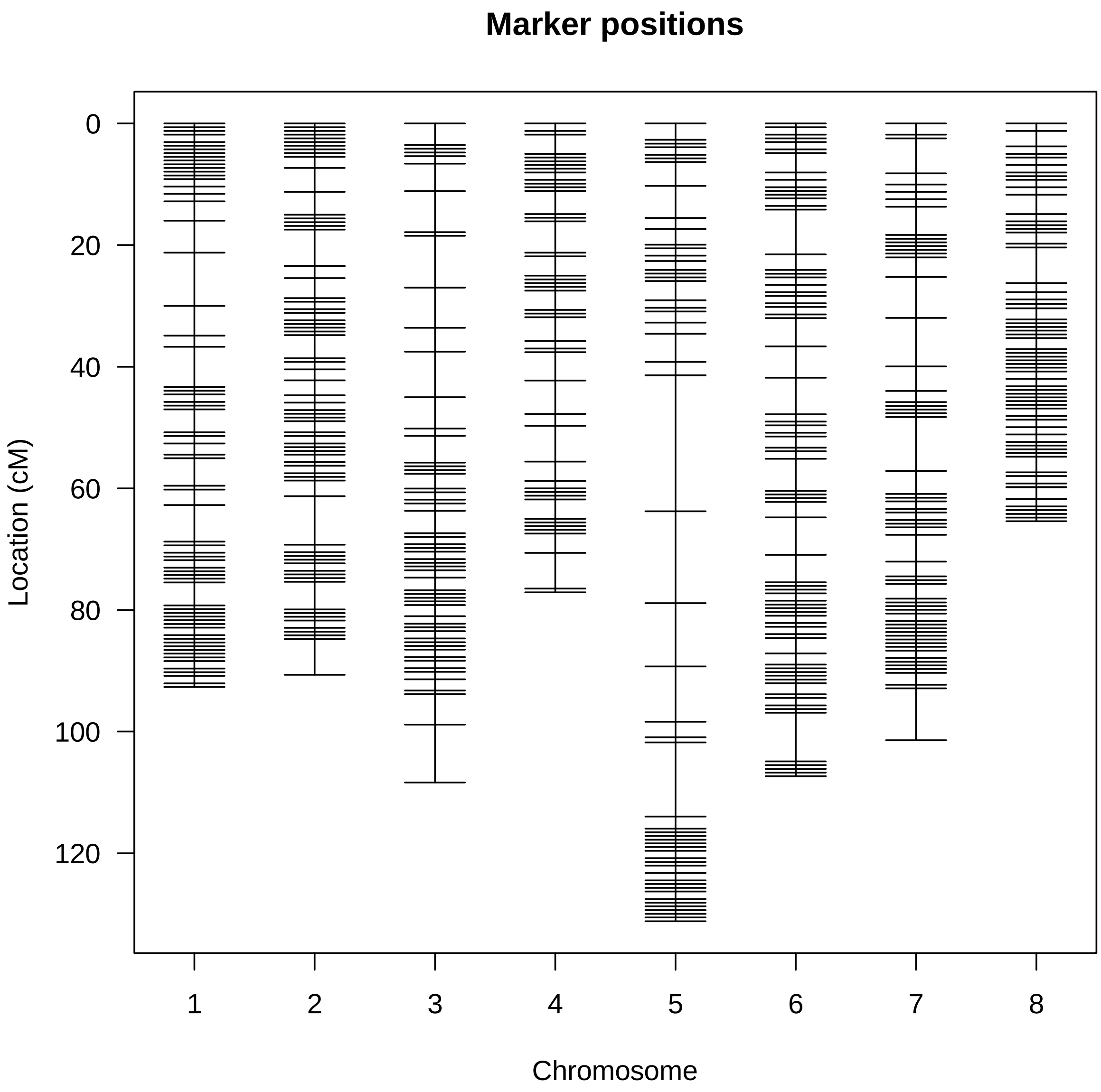

Figure S1. Distribution of 501 genetic markers in the *Erysimum cheiranthoides* Konstanz x Elbtalaue F2 population genetic map. Figure was generated using the plotMap() function in R/qtl.

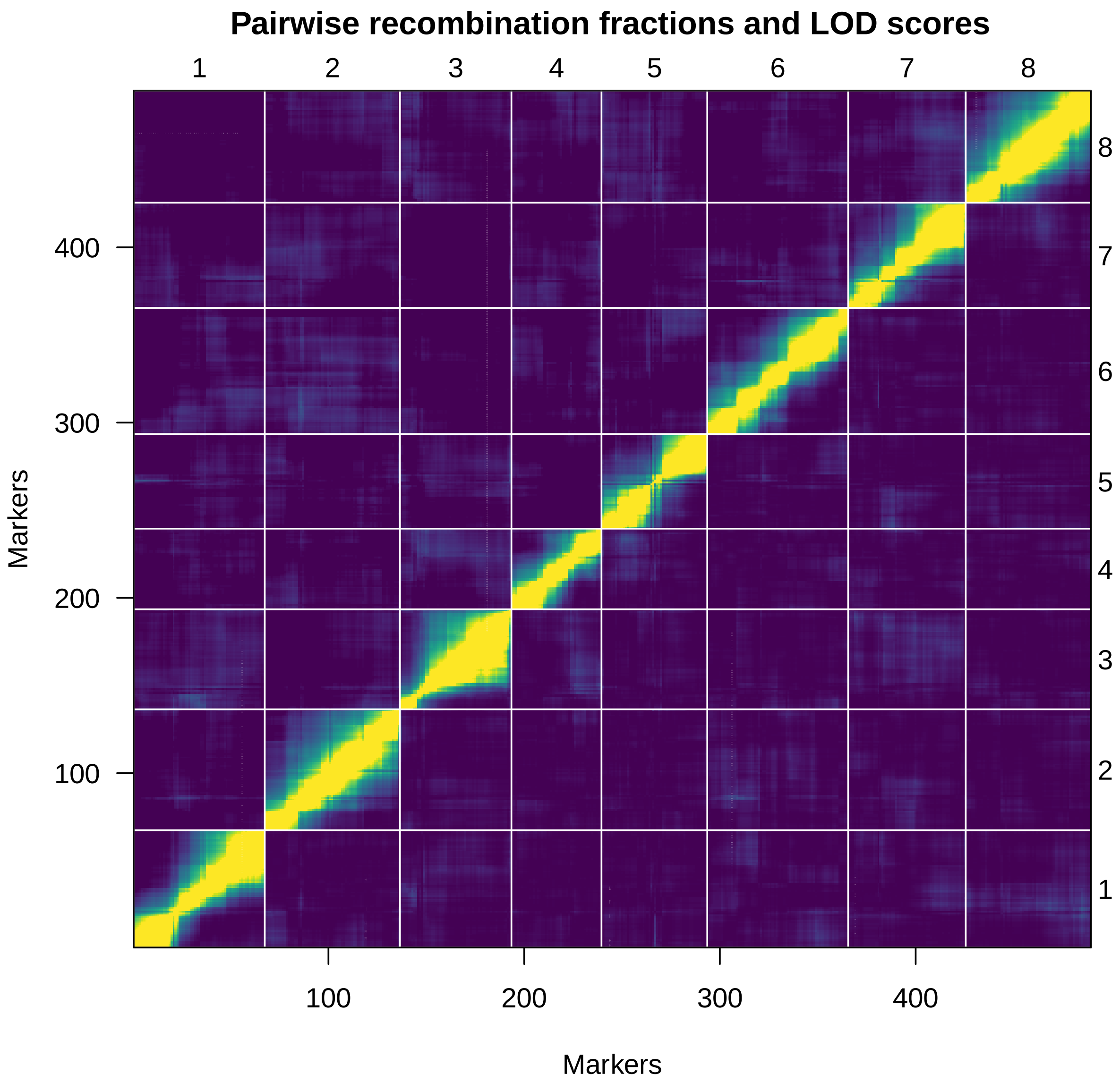

Figure S2. Genetic linkage of *Erysimum cheiranthoides* chromosomal markers. Pairwise recombination fractions (above diagonal) and LOD scores (below diagonal) indicating probability of genetic linkage for 501 genetic markers in the Konstanz x Elbtalaue genetic map. The 8 linkage groups correspond to the 8 chromosomes of *E. cheiranthoides*. Figure was generated using the plotRF() function in R/qtl.

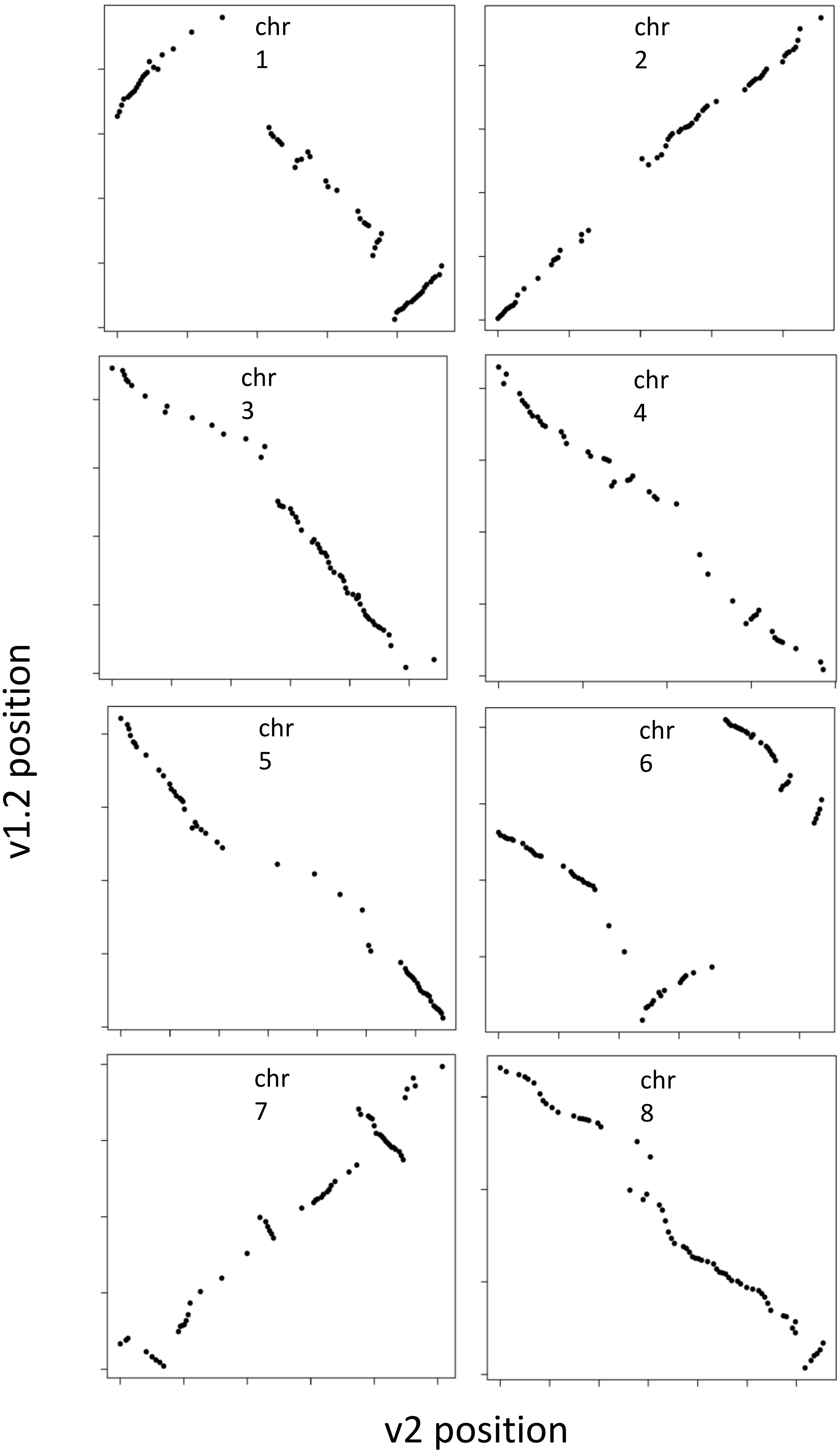

Figure S3. Position of genetic markers in *Erysimum cheiranthoides* genome v1.2 (Hi-C proximity guided assembly) and v2.0 (assembled using a classical genetic map). Large regions on chromosomes 1, 4, 6, 7, and 8 were inverted or incorrectly ordered in v1.2.

	E. cheiranthoides **v1.2**	*E. cheiranthoides* **v2.0**
**Total length (Mbp)**	177.2	174.1
**Number of chromosomes**	8	8
**Chloroplast assembly**	no	yes
**N50 (Mbp)**	22.4	21.9
**% of assembly anchored on chromosomes**	98.5	99.0
**Number of unanchored contigs**	216	123

Fiugre S4. Comparison of assembly statistics for *Erysimum cheiranthoides* genome v1.2 and v2.0. In addition to improved congruency with genetic mapping data (Figure 3), v2.0 includes a chloroplast assembly and fewer unanchored contigs. It is unclear why v2.0 is 3.1 Mbp shorter than v1.2, but the total length of v2.0 matches the total length of the PacBio contigs.

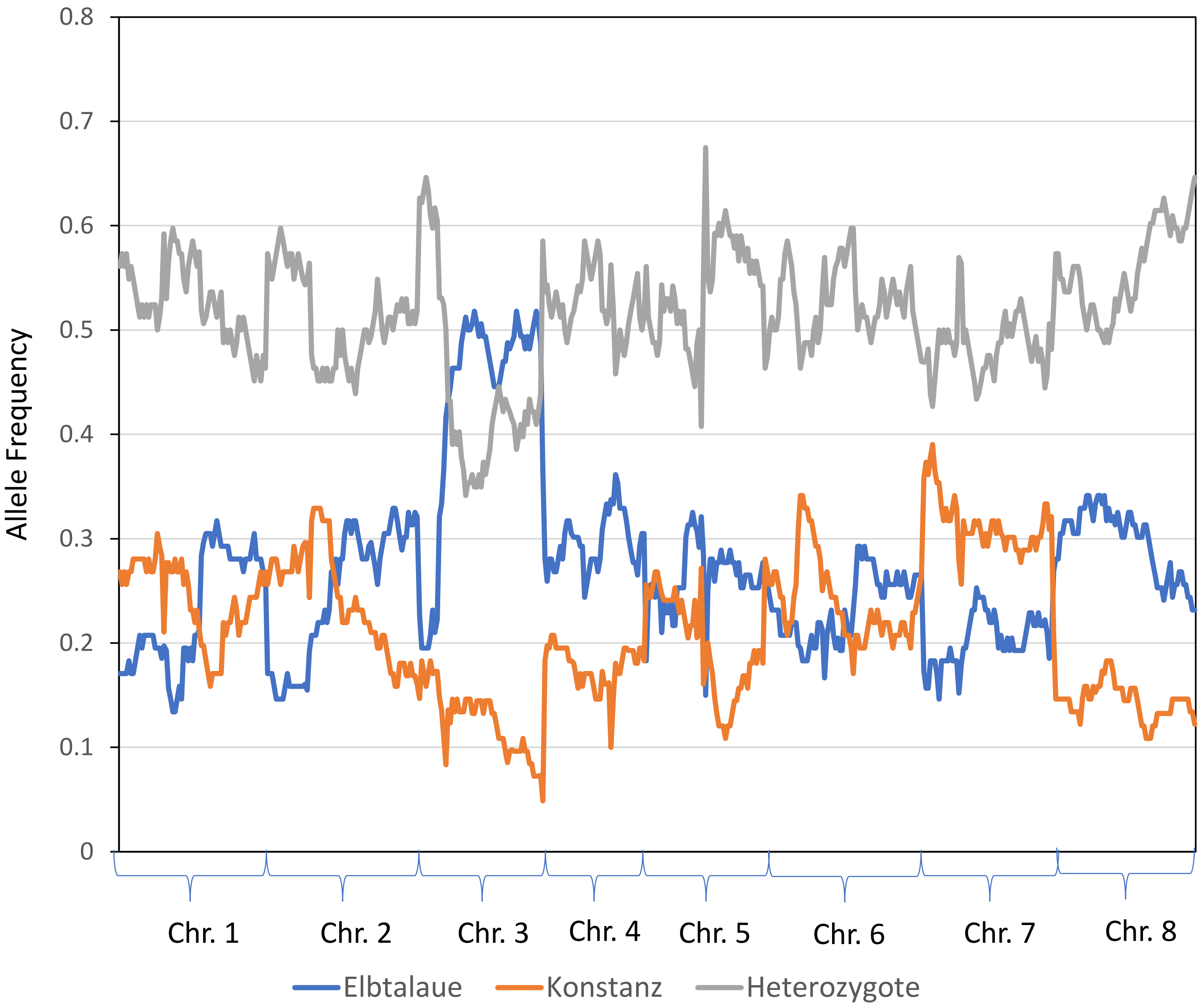

Figure S5. Allele frequencies across the *Erysimum cheiranthoides* F2 mapping population. The frequency of homozygous Elbtalaue, homozygous Konstanz, and heterozygous alleles for 501 molecular markers across 83 F2 lines is shown.

A

```
Bo_CYP79F1              MTMMMILTTSLPYPFQILLVFILSMASITLLGRILSRPTKTKDRSRQLPPGPPGWPILGN   60
AT1G16400               ----MMMKISFNTCFQILLGFIVFIASITLLGRIFSRPSKTKDRCRQLPPGRPGWPILGN   56
Bs_BCMA2                -MMMMSLTTSLPYPFQILLVFIVSMASIYILGRILSRPTKTKDRSRQLPPGPPGWPILGN   59
Bs_BCMA1                -MMMMSLTTSLPYPFQILLVFIVSMASISILGRILSRPTKAKDRSRHLPPSPPEWPILGN   59
Bs_BCMA3                -MMMMSLTTSLPYPFQILLVFIVSMASISILGRILSRPTKTKDRSRHLPPSPPEWPILGN   59
AT1G16410               ---MMSFTTSLPYPFHILLVFILSMASITLLGRILSRPTKTKDRSCQLPPGPPGWPILGN   57
Erche01g017900_Elb      MMMMMSLSTSLTYPFQILLVFVVSMASISLLRRILSKPTKTKDRSRQLPPGPPGWPILGN   60
Erche01g017900_Kon      MMMMMSLSTSLTYPFQILLVFVVSMASISLLRRILSKPTKTKDRSRQLPPGPPGWPILGN   60
                            * :. *:   *:*** *:: :*** :* **:*:*:*:***. :***[illegible]* ******

Bo_CYP79F1              LPELMMTRPRHKYVDIAMKGQKPDIACFNFAGTHAIIINSDEIAREALKERDADFADRPN   120
AT1G16400               LPELIMTRPRSKYFHLAMKELKTDIACFNFAGTHTITINSDEIAREAFRERDADLADRPQ   116
Bs_BCMA2                LPELFMTRPRSKYFHLVTKELKTDITCFNFAGVRAITINSDEIAREAFRERDADLADRPP   119
Bs_BCMA1                LPELFMTRPRSKYFDLAMKELKTDIACFNFAGVRAITIKSVEIAREAFRERDADLADRPH   119
Bs_BCMA3                LPELFMTRPRSKYFDLAMKELKTDIACFNFAGVRAIIINSVEIAREAFRERDADLADRPH   119
AT1G16410               LPELFMTRPRSKYFRLAMKELKTDIACFNFAGIRAITINSDEIAREAFRERDADLADRPQ   117
Erche01g017900_Elb      LPELFMTRPRAKYFRLAMKELKTDIACFNFAGVHAITIHSDEIAREAFRERDAELADRPN   120
Erche01g017900_Kon      LPELFMTRPRAKYFRLAMKELKTDIACFNFAGVHAITIHSDEIAREAFRERDAELADRPN   120
                        ****:***** **. :. *  * **:****** ::* *:* ******::****::****

Bo_CYP79F1              LFNMRTIGGNHKSMGNSPYGEQFMKMKRVITTEIMSVKTLNMLVAARTIEADNLLAYLLS   180
AT1G16400               LSIVESIGDNYKTMGTSSYGEHFMKMKKVITTEIMSVKTLNMLEAARTIEADNLIAYIHS   176
Bs_BCMA2                LFMIQTIGDNYKSMGSAPYGGQFMKMKKVITTEIMSVKTLNMMVAARTIEADNLIAYVHS   179
Bs_BCMA1                LFIMETIGDNYKSMIVSPYGEQFMKMKRVITTEIMSVKTLTMLAAARSIEADNLIAYVHS   179
Bs_BCMA3                LFIMETIGDNYKSMLISPYGEQFMKMKRVITTEIMSVKTLNMLAAARSIEADNLIAYVHS   179
AT1G16410               LFIMETIGDNYKSMGISPYGEQFMKMKRVITTEIMSVKTLKMLEAARTIEADNLIAYVHS   177
Erche01g017900_Elb      LFIMETIGDNYKSMGISPYGEQFMKMKRVITTEIMSVKTLNMLVAARTIEADNLIAYVHS   180
Erche01g017900_Kon      LFIMETIGDNYKSMGISPYGEQFMKMKRVITTEIMSVKTLNMLVAARTIEADNLIAYVHS   180
                        *  :.:**.*:*:*[illegible]: ** :*****:************.*: ***:******:**: *

Bo_CYP79F1              MYKRSETADVREFSRVYGYAVTMRLLFGRRHVTKENVFSDEGRLGQAEKDHLDAIFETLN   240
AT1G16400               MYQRSETVDVRELSRVYGYAVTMRMLFGRRHVTKENMFSDDGRLGKAEKHHLEVIFNTLN   236
Bs_BCMA2                MYQRSETVDVRELSRVYGYAVTMRMLFGRRHVTKENVFSDEGRLGKAERHHLEAIFNTLN   239
Bs_BCMA1                MYQRSETVDVRELSRVYGYAVTMRMLFGRRHVTKENVFSDEGRLGKAEKHHLEAIFNTLN   239
Bs_BCMA3                MYQRSETVDVRELSRVYGYAVTMRMLFGRRHVTKENVFSDEGRLGKAEKHHLEAIFNTLN   239
AT1G16410               MYQRSETVDVRELSRVYGYAVTMRMLFGRRHVTKENVFSDDGRLGNAEKHHLEVIFNTLN   237
Erche01g017900_Elb      MYQRSETVDVRELSRVYGYTVTMRMLFGRRHVTKENVYSDDGRLGKAEKHHLDAIFNTLN   240
Erche01g017900_Kon      MYQRSETVDVRELSRVYGYTVTMRMLFGRRHVTKENVYSDDGRLGKAEKHHLDAIFNTLN   240
                        **:****.****:******:****:***********::**:****:**:.**:.**:***

Bo_CYP79F1              CLPSFSPADYLEKWFRGWNIDGQEERVVMYCNKVRSYNNPIIDERVELWREKGGKAAVED   300
AT1G16400               CLPGFSPVDYVDRWLGGWNIDGEEERAKVNVNLVRSYNNPIIDERVEIWREKGGKAAVED   296
Bs_BCMA2                SLPSFSPTDYMERWLKGWNIGGQEERVTVNCNIVRSYNNPIVDERVELWREKGGKAAVED   299
Bs_BCMA1                CFPSFSPADYVERWLKGWNIDGQEERVTVNCNIVRSYNNPIIDERVELWREKGGKAVVED   299
Bs_BCMA3                CLPSFSPADYVERWLKGWNIDGQEERVTMNCNIVRSYNNPIIDERVELWREKGGKAVVED   299
AT1G16410               CLPSFSPADYVERWLRGWNVDGQEKRVTENCNIVRSYNNPIIDERVQLWREEGGKAAVED   297
Erche01g017900_Elb      CLPAFSPADYVERWLRGWNVDGQEQRVKVNCNIVRSYNNRIIDERVELWREKGGKAAVED   300
Erche01g017900_Kon      CLPAFSPADYVERWLRGWNVDGQEQRVKVNCNIVRSYNNRIIDERVELWREKGGKAAVED   300
                        .:*.***.**:::*: ***:.*:*:*.    * ****** *:****::***:****.***

Bo_CYP79F1              WIDTFITLKDENGKYYITPDEVKAQCVEFCIAAIDNPANNMEWTLAEMLKNPEILKKALK   360
AT1G16400               WLDTFITLKDQNGNYLVTPDEIKAQCVEFCIAAIDNPANNMEWTLGEMLKNPEILRKALK   356
Bs_BCMA2                WLDTFITLKDQNGNYMVTPDEIKAQYVEFCIAAIDNPANNMEWTLAEMLKNPEILRKALE   359
Bs_BCMA1                WLDTFITLKDQNGKYVVTPDEIKAQCVEFCIAAFDNPANNMEWTLAEMLKNPEILRKALK   359
Bs_BCMA3                WLDTFITLKDQNGKYVVTPDEIKAQCVEFCIAAFDNPANNMEWTLAEMLKNPEILKKALK   359
AT1G16410               WLDTFITLKDQNGKYLVTPDEIKAQCVEFCIAAIDNPANNMEWTLGEMLKNPEILRKALK   357
Erche01g017900_Elb      WLDTFITLKDQNGKYLVTPDEIKAQCVEFCIAAIDNPANNMEWTLAEMLKNPEILKKALK   360
Erche01g017900_Kon      WLDTFITLKDQNGKYLVTPDEIKAQCVEFCIAAIDNPANNMEWTLAEMLKNPEILKKALK   360
                        *:********:**:* :****:*** *******:***********.*********:***:
```

```
Bo_CYP79F1              ELDEVVGRDRLVQESDIPNLNYLKACCRETFRIHPSAHYVPTHVARQDTTLGGYFIPKGS    420
AT1G16400               ELDEVVGKDRLVQESDIRNLNYLKACCRETFRIHPSAHYVPPHVARQDTTLGGYFIPKGS    416
Bs_BCMA2                ELEEVVGKDRLVQESDIPNLNYLKACCRETFRIHPSAHYVPPHVARQDTTLGGYFIPKGS    419
Bs_BCMA1                ELDEVVGKDRLVQESDIPNLNYLKACCRETFRIHPSSHYAPPHVARQDTTLGGYFIPKGS    419
Bs_BCMA3                ELDEVVGKDRLVQESDIPNLNYLKACCRETFRIHPSTHYVPTHVARQDTTLGGYFIPEGS    419
AT1G16410               ELDEVVGRDRLVQESDIPNLNYLKACCRETFRIHPSAHYVPSHLARQDTTLGGYFIPKGS    417
Erche01g017900_Elb      ELDEVVGKDRLVQESDIPNLTYLKACCRETFRIHPSAHYVPPHVARQDTTLGGYFIPKGS    420
Erche01g017900_Kon      ELDEVVGKDRLVQESDIPNLTYLKACCRETFRIHPSAHYVPPHVARQDTTLGGYFIPKGS    420
                        **:****:********* **.***************:**.* *:*************:**

Bo_CYP79F1              HIHVGRPGIGRSSKIWTDPLVYKPERHLEGDGISKELSLVETELRFVSFGTGRRGCVGVK    480
AT1G16400               HIHVCRPGLGRNPKIWKDPLAYEPERHLQGDGITKEVTLVETEMRFVSFSTGRRGCVGVK    476
Bs_BCMA2                HIHVCRPGLGRNPKIWKDPLVYQPERHLQGDRITKEVTLVEAEMRFVSFSTGRRGCIGVK    479
Bs_BCMA1                HIHVCRRGLGQNPKIWKDPLLYKPERHLQGDGITQEVTLVETEMRFVSFGTGRRGCIGVK    479
Bs_BCMA3                HIHVCRRGLGQNPKIWKDPLVYKPERHLQGDGITQEVTLVETEMRFVSFGTGRRGCIGVK    479
AT1G16410               HIHVCRPGLGRNPKIWKDPLVYKPERHLQGDGITKEVTLVETEMRFVSFSTGRRGCIGVK    477
Erche01g017900_Elb      HIHVGRPGLGRNPKIWKDPLVYKPERHLQGDGITKEVTLVETEMRFVSFSTGRRGCIGVK    480
Erche01g017900_Kon      HIHVGRPGLGRNPKIWKDPLVYKPERHLQGDGITKEVTLVETEMRFVSFSTGRRGCIGVK    480
                        **** * *:*:. ***.*** *:*****:** *::*::***:*:*****.******:***

Bo_CYP79F1              VGTIMMVIMLARFLQAFNWKLHPGYGPLSLEEDDA-LLMAKPLLLSVEPRLAPNLYPKFC    539
AT1G16400               VGTIMMAMMLARFLQGFNWKLHRDFGPLSLEEDDASLLMAKPLLLSVEPRLASNLYPKFR    536
Bs_BCMA2                VGTIMMVMMLARFIQGFNWKLHQDFGPLSLEEDDESLLMAKPLLLSVEPRLAPNLYPKFR    539
Bs_BCMA1                VGTIMMVMMLARFIQGFNWKLHQDFGPLSLEEDDASLLMAKPLLVSVELRLAPHFYKKIR    539
Bs_BCMA3                VGTIMMVMMLARFIQGFNWKLHQDFGPLSLEEDDASLLMAKPLLLSVEPRLAPNLYKKFR    539
AT1G16410               VGTIMMVMLLARFLQGFNWKLHQDFGPLSLEEDDASLLMAKPLHLSVEPRLAPNLYPKFR    537
Erche01g017900_Elb      VGTIMMVMMLARFLQAFNWKLHQDFGPLSLEEDDA-LLMAKPLLLSVEPRLAPNLYPKFR    539
Erche01g017900_Kon      VGTIMMVMMLARFLQAFNWKLHQDFGPLSLEEDDA-LLMAKPLLLSVEPRLAPNLYPKFR    539
                        ******.::****:*.****** .:*********  ******* :*** *** ::*.*:

Bo_CYP79F1              P       540
AT1G16400               P       537
Bs_BCMA2                H       540
Bs_BCMA1                P       540
Bs_BCMA3                P       540
AT1G16410               P       538
Erche01g017900_Elb      P       540
Erche01g017900_Kon      P       540
```

B

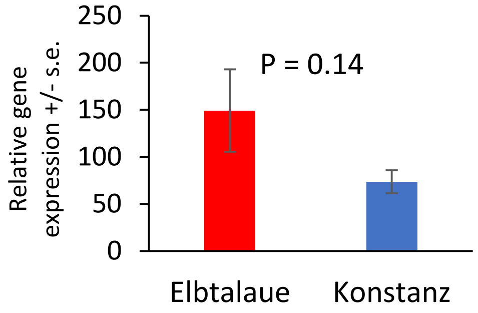

Figure S6. Alignment of CYP79F sequences and gene expression. (A) CYP79F protein sequences from *Erysimum cheiranthoides* (Erche), *Arabidopsis thaliana* (AT), *Boechera stricta* (Bs), and *Brassica oleracea* (Bo) were aligned using Clustal Omega (https://www.ebi.ac.uk/Tools/msa/clustalo/). Red arrows indicate positions with amino acid variation in *B. stricta* that has been associated with the differences in the preferential incorporation of methionine (BCMA2) or branched chain amino acids (BCMA1 and BCMA3) into glucosinolates side chains (Prasad et al, 2012, *Science* 337:1081-1084). The *E. cheiranthoides* Elbtalaue (Elb) and Konstanz (Kon) alleles differ at one of these positions, having glycine and serine, respectively at position 51. (B) Expression levels of Erche01g017900 in the Elbtalaue and Konstanz accessions. Mean +/- s.e. of N = 5; P value is from a two-tailed *t*-test.

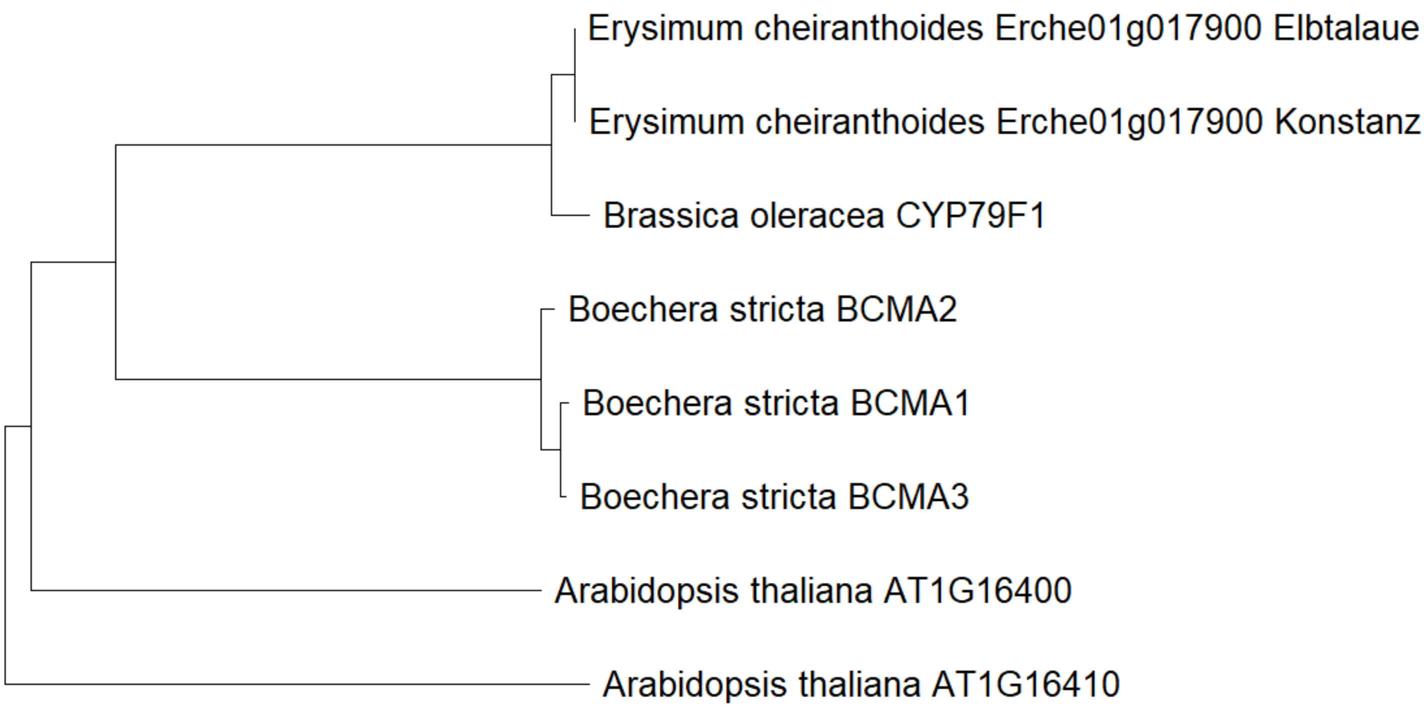

Figure S7. Neighbor-joining tree of CYP79F protein sequences. CYP79F protein sequences from *Erysimum cheiranthoides*, *Arabidopsis thaliana*, *Boechera stricta*, and *Brassica oleracea* were aligned using the Muscle algorithm and a neighbor-joining tree was constructed using MEGA11.

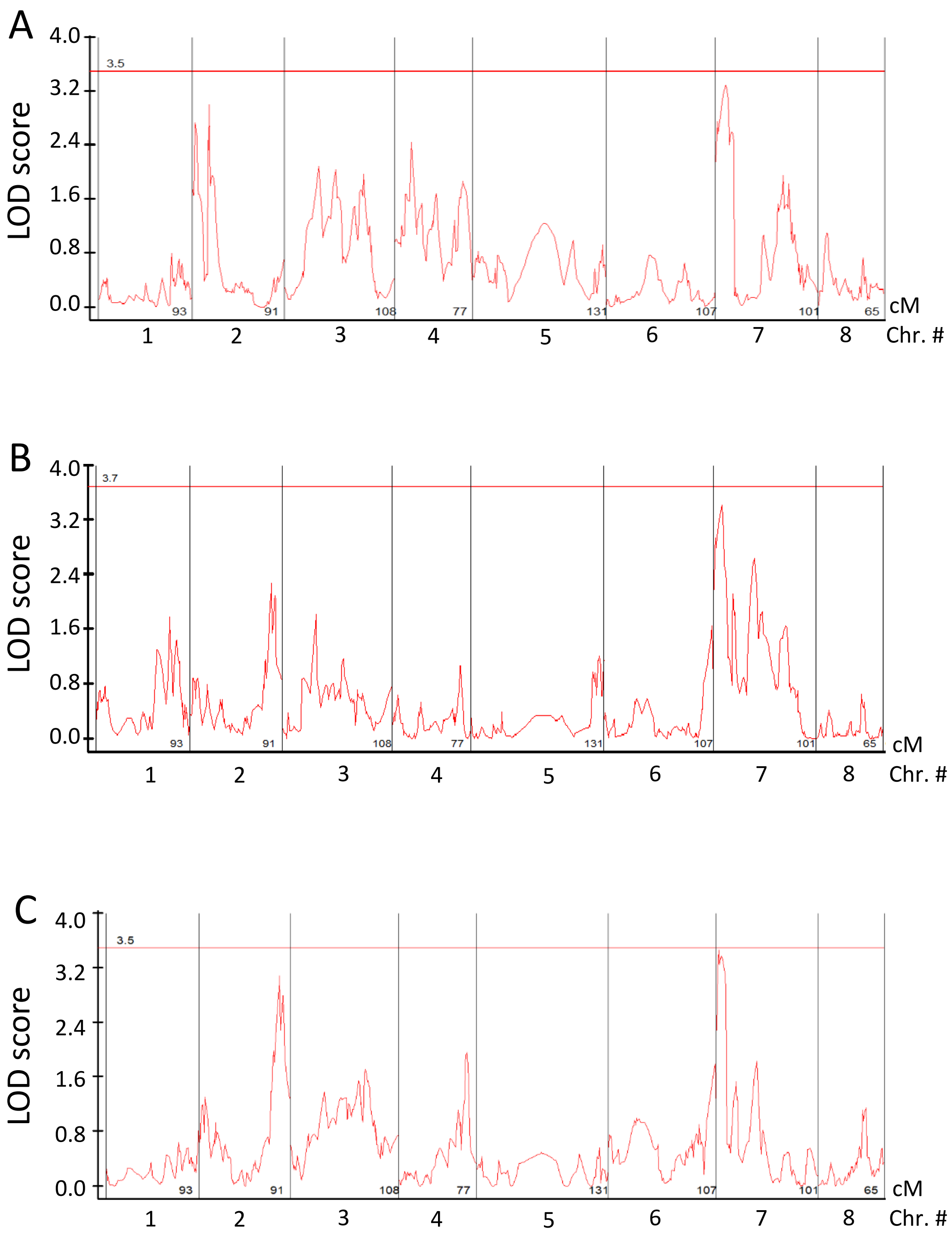

Figure S8. Quantitative trait loci (QTL) affecting glucosinolate abundance in *Erysimum cheiranthoides*. LOD plots of (A) 3-methylsulfinylpropylglucosinolate (3MSIP), (B) 3-methylsulfonylpropylglucosinolate (3MSOP), and (C) 4-methylsulfonylbutylglucosinolate (4MSOB) abundance in an Elbtalaue x Konstanz F2 population are shown. Horizontal lines in are 95% confidence levels, calculated based on 500 permutations of the data. Graphs were created using Windows QTL Cartographer.

```
AT5G53810_IGMT5      MANHLQVPLTKPDRVKEEQEVEEEARLLARRLANAAASPMVLKAALELGVIDTITTV-GG    59
R_sativus_           MGFPFEETLSSNPKTQTVVDDDNELGLMAVRLANAAAFPMVLKASLELGVFDTLHAEAAR    60
B_oleracea_          MGILIEETLSANSNTQTVIDDDNELGLMAVRLANAAAFPMVLKAALELGVFDTLYAEAAR    60
Erche01g022144       MGYLFEETVSSNPKTTIVTDDDNELGLMAVRLANAAAFPMVLKAALELGVFDTLYAEASR    60
Erche01g022140       MGFLFEESLSSNSKTPIVVDDDNQLGLMAVRLANAAAFPMVLKAALELGVFDTLYAETSS    60
Erche01g022141       MGFLFEESLSSNSKTPIVVDDDNQLGLMAVRLANAAAFPMVLKAALELGVFDTLYAEASR    60
AT1G21130_IGMT4      MGYLLEETLSSNSKTPIVIDDDNELGLMAVRLANAAAFPMVLKAALELGVFDTLYAEASR    60
AT1G21100_IGMT1      MGYLFQETLSSNPKTPIVVDDDNELGLMAVRLANAAAFPMVLKAALELGVFDTLYAAASR    60
AT1G21120_IGMT2      MGYLFEETLSSNPKTPIVVDDDNELGLMAVRLANAAAFPMVLKASLELGVFDTLYAEASR    60
AT1G21110_IGMT3      MGYLFEETLSSNPKTPIVVDDDNELGLMAVRLANAAAFPMVLKASLELGVFDTLYAEASR    60
                     *.  ::  ::   ..    : :::  *:* ******* ******:*****:**: :  .

AT5G53810_IGMT5      GDLWLSPSEIALRLPTKPCNLEAPALLDRMLRFLVSHSVLKCRTVIEENGQTGKVERVYA    119
R_sativus_           TDAFLSPSEIASRLPTTPRNPEAPVLLDRMLRLLASYSMVKCDKA-------GKGERTYR    113
B_oleracea_          TNSFLSPSEIASRLPTTPRNPEAPVLLDRMLRLLASYSMVKCGKVSS-----GKVERVYR    115
Erche01g022144       TDSFLSPSEIASKLPTTPRNPEAPVLLDRMLRLLASYSMVKCGTISA-----GKGERVYR    115
Erche01g022140       TDSFLSPSEIASKLPTTPSNPEAPVLLDRMLRLLASYSMVKCGKVLE-----GKGERVYK    115
Erche01g022141       TDSFLSPSEIASKLPTTPRNPEAPVLLDRMLRLLASYSMVKCGKVLE-----GKGERVYK    115
AT1G21130_IGMT4      SDSFLSPSEIASKLPTTPRNPEAPVLLDRMLRLLASYSVVKCGKVSE-----GKGERVYR    115
AT1G21100_IGMT1      TDSFLSPYEIASKLPTTPRNPEAPVLLDRMLRLLASYSMVKCGKALS-----GKGERVYR    115
AT1G21120_IGMT2      TDSFLSPSEIASKLPTTPRNPGAPVLLDRMLRLLASYSMVKCEKVSV-----GKGERVYR    115
AT1G21110_IGMT3      TDSFLSPSEIASKLPTTPRNPGAPVLLDRMLRLLASYSMVKCEKVSV-----GKEQRVYR    115
                      : :*** *** :***.* *  **.*******:*.*:*::** .        ** :*.*

AT5G53810_IGMT5      AEPVCKYLLNKSDDVSGSFASLFMLDLSDVFIKTWTHLEDVILEGRDAFSSAH-GMKLFE    178
R_sativus_           AEPICRFFLKDNIQDIGSLASQVIVNFDSVFLNTWAQLKDVVLEGGDAFGRAHGGMKLFD    173
B_oleracea_          AEPICRFFLKDNIQDIGSLASQVIVNFDSVFLNTWAQLKDVVLEGGDAFGRAHGGMKLFD    175
Erche01g022144       AEPICRFFLKDNIQDIGSLASQVIVNFDSVFLNTWAQLKDVVLEGGDAFGRAHGGMKLFD    175
Erche01g022140       AEPICRFFLKNNIQDIGSLASQVIVNFDSVFLNTWAQLKDVVLEGGDAFGRAHGGMKLFD    175
Erche01g022141       AEPICRFFLKDNIQDIGSLASQVIVNFDSVFLNTWAQLKDVVLEGGDAFGRAHGGMKLFD    175
AT1G21130_IGMT4      AEPICRFFLKDNIQDIGSLASQVIVNFDSVFLNTWAQLKDVVLEGGDAFGRAHGGMKLFD    175
AT1G21100_IGMT1      AEPICRFFLKDNIQDIGSLASQVIVNFDSVFLNTWAQLKDVVLEGGDAFGRAHGGMKLFD    175
AT1G21120_IGMT2      AEPICRFFLKNNIQDIGSLASQVIVNFDSVFLNTWAQLKDVVLEGGDAFGRAHGGMKLFD    175
AT1G21110_IGMT3      AEPICRFFLKNNIQDIGSLASQVIVNFDSVFLNTWAQLKDVVLEGGDAFGRAHGGMKLFD    175
                     ***:*:::*:.. :  **:** .::::..**::**::*:**:*** ***. ** *****:

AT5G53810_IGMT5      YIQADERFGKVFNRAMLESSTMVTEKVLKFYEGFKDVKTLVDVGGGLGNTLGLITSKYPH    238
R_sativus_           YMGTDERFSKLFNQTGFT--IAVVKKALEVYQGFEDVDVLVDVGGGVGNTLGVVTSKYPN    231
B_oleracea_          YMGTDERFSKLFNQTGFT--IAVVKKALEVYQGFKDVNVLVDVGGGVGNTLGVVTSKYPN    233
Erche01g022144       YMGTDERFSKLFNQTGFT--IAVVKKALEVYEGFKGVNVLVDVGGGVGNTLGVVTSKYPN    233
Erche01g022140       YMGTDERFSKLFNQTGFT--IAVVKKALEVYEGFKGVNVLVDVGGGVGNTLGVVTSKYPN    233
Erche01g022141       YMGTDERFSKLFNQTGFT--IAVVKKALEVYEGFKGVNVLVDVGGGVGNTLGVVTSKYPN    233
AT1G21130_IGMT4      YMGTDERFSKLFNQTGFT--IAVVKKALEVYQGFKGVNVLVDVGGGVGNTLGVVASKYPN    233
AT1G21100_IGMT1      YMGTDERFSKLFNQTGFT--IAVVKKALEVYEGFKGVKVLVDVGGGVGNTLGVVTSKYPN    233
AT1G21120_IGMT2      YMGTDERFSKLFNQTGFT--IAVVKKALEVYQGFKGVNVLVDVGGGVGNTLGVVTSKYPN    233
AT1G21110_IGMT3      YMGTDERFSKLFNQTGFT--IAVVKKALEVYQGFKGVNVLVDVGGGVGNTLGVVTSKYPN    233
                     *: :****.*:**:: :     *.:*.*:.*:**:.*..*******:*****:::****:

AT5G53810_IGMT5      LIGINFDLAPVLANAHSYPGVNHVAGDMFIKIPKGDAIFMKWILHDWTDEQCVAILKNCW    298
R_sativus_           IKGINFDLTCALAQAPSYPGVEHVAGDMFVDVPKGDAMILKRILHDWTDEDCVKILKNCW    291
B_oleracea_          IKGINFDLTCALAQAPSYPGVEHVPGDMFVDVPKGDAMILKRILHDWTDEDCVKILKNCW    293
Erche01g022144       IRGINFDLTCALAQARSYPGVEHVAGDMFVDIPTGDAMILKRILHDWTDEDCVKILKNCW    293
Erche01g022140       IKGINFDLICALEQAPSYPGVEHVAGDMFVDVPTGDAMILKRILHDWTDEDCVKILKNCW    293
Erche01g022141       IKGINFDLICALEQAPSYPGVEHVAGDMFVDVPTGDAMILKRILHDWTDEDCVKILKNCW    293
AT1G21130_IGMT4      IKGINFDLTCALAQAPSYPGVEHVAGDMFVDVPTGDAMILKRILHDWTDEDCVKILKNCW    293
AT1G21100_IGMT1      IKGINFDLTCALAQAPSYPGVEHVAGDMFVDVPTGDAMILKRILHDWTDEDCVKILKNCW    293
AT1G21120_IGMT2      IKGINFDLTCALAQAPSYPGVEHVAGDMFVDVPTGDAMILKRILHDWTDEDCVKILKNCW    293
AT1G21110_IGMT3      IKGINFDLTCALAQAPTYPGVEHVAGDMFVDVPTGNAMILKRILHDWTDEDCVKILKNCW    293
                     : ******  .* :* :****:** ****:.:*.*:*:::* ********:** ******
```

```
AT5G53810_IGMT5     KSLEENGKLIIVEMVTPVEAKSGDICSNIVFGMDMTMLTQCSGGKERDLYEFENLAYASG     358
R_sativus_          KSLPENGKVVVIELVTPDDAENGDINANIAFDMDMLMFTQCSGGKERSRAEFEALAAASG     351
B_oleracea_         KSLPENGKVVVIELVTPDDAENGDINANIAFDMDMLMFTQCSGGKERSRAEFEALAMEAG     353
Erche01g022144      KSLPENGKVVVIELVTPDDAENGDINANIAFDMDMLMFTQCSGGKERSRAEFEALAAASG     353
Erche01g022140      KSLPENGKVVVIELVTPDDAENGDINANIAFDMDMLMFTQCSGGKERSRAEFEALAAASG     353
Erche01g022141      KSLPENGKVVVIELVTPDDAENGDINANIAFDMDMLMFTQCSGGKERSRAEFEALAAASG     353
AT1G21130_IGMT4     KSLPESGKVVVIELVTPDEAENGDINANIAFDMDMLMFTQCSGGKERSRAEFEALAAASG     353
AT1G21100_IGMT1     KSLPENGKVVVIELVTPDEAENGDINANIAFDMDMLMFTQCSGGKERSRAEFEALAAASG     353
AT1G21120_IGMT2     KSLPENGKVVVIELVTPDEAENGDINANIAFDMDMLMFTQCSGGKERSRAEFEALAAASC     353
AT1G21110_IGMT3     KSLPQNGKVVVIELVTPDEAENGDINANIAFDMDMLMFTQCSGGKERSRAEFEALAAASG     353
                    *** :.**::::*:*** :*:.*** :**.*.*** *:*********.  *** **  : 

AT5G53810_IGMT5     FSRCAIVCAVYPFSVIEIYK---    378
R_sativus_          FTHCKFVCQAYHCWIIEFCKENA    374
B_oleracea_         FTHCKFVCQAYHCWIIEFCK---    373
Erche01g022144      FTHCNFVCQAYHCWIIEFCK---    373
Erche01g022140      FTHCKFVCQAYHCWIIEFCK---    373
Erche01g022141      FTHCKFVCQAYHCWIIEFCKGNV    376
AT1G21130_IGMT4     FTHCKFVCQAYHCWIIEFCK---    373
AT1G21100_IGMT1     FTHCKFVCQAYHCWIIEFCK---    373
AT1G21120_IGMT2     FTHCKFVCQAYHCWIIEFCK---    373
AT1G21110_IGMT3     FSHCQFVCQAYHCWIIEFCK---    373
                    *::* :** .*   :**: *
```

Figure S9. Alignment of indole glucosinolate methyltransferase (IGMT) protein sequences. **S**equences of predicted IGMT proteins from *Erysimum cheiranthoides* (Erche01g022140.a, Erche01g022140.b, and Erche01g022140.d), *Arabidopsis thaliana* (AT1G21100, AT1G21110, AT1G21120, AT1G21130, and AT5G53810), radish (*Raphanus sativus*), and cabbage (*Brassica oleracea*) were aligned using Clustal Omega (https://www.ebi.ac.uk/Tools/msa/clustalo/)

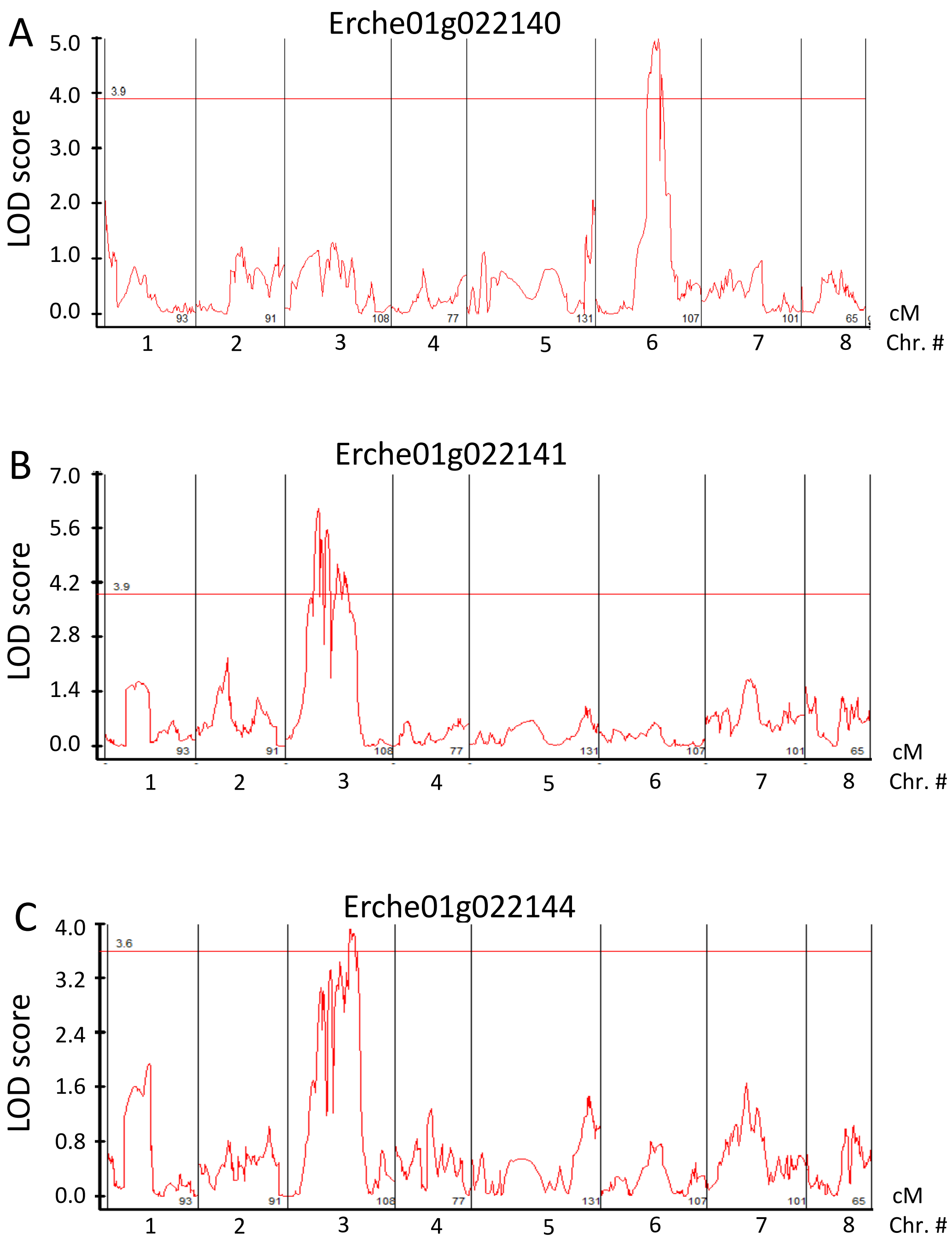

Figure S10. Quantitative trait loci (QTL) affecting indole glucosinolate methyltransferase gene expression in *Erysimum cheiranthoides*. LOD plots of (A) Erche01g022140.a, (B) Erche01g022140.b, and (C) Erche01g022140.c expression levels in an Elbtalaue x Konstanz F2 population are shown. Horizontal lines in are 95% confidence levels, calculated based on 500 permutations of the data. Graphs were created using Windows QTL Cartographer.
